# Congenital absence of the left pericardium presenting as cardiogenic shock: a case report

**DOI:** 10.1093/ehjcr/ytag308

**Published:** 2026-05-08

**Authors:** Juliette Piccoli, Elodie Phamisith, Marine Duchaine, Fabrice Vanhuyse, Juan-Pablo Maureira

**Affiliations:** Department of Cardiovascular Surgery and Heart Transplantation, University Hospital of Nancy-Brabois, Rue du Morvan, Vandœuvre-lès-Nancy 54500, France; Department of Cardiovascular Surgery and Heart Transplantation, University Hospital of Nancy-Brabois, Rue du Morvan, Vandœuvre-lès-Nancy 54500, France; Department of Radiology, University Hospital of Nancy-Brabois, Rue du Morvan, Vandoeuvres-lès-Nancy 54500, France; Department of Cardiovascular Surgery and Heart Transplantation, University Hospital of Nancy-Brabois, Rue du Morvan, Vandœuvre-lès-Nancy 54500, France; Department of Cardiovascular Surgery and Heart Transplantation, University Hospital of Nancy-Brabois, Rue du Morvan, Vandœuvre-lès-Nancy 54500, France

**Keywords:** Congenital absence of pericardium, Heart’s levorotation, Cardiogenic shock, Case report

## Abstract

**Background:**

Congenital absence of the pericardium is a rare and often incidental finding, but it may present with severe cardiovascular compromise in exceptional situations.

**Case summary:**

A 65-year-old man presented with cardiogenic shock associated with diffuse ST-segment elevation on electrocardiogram and severe metabolic acidosis. Computed tomography imaging excluded aortic dissection but revealed marked cardiac levorotation suggestive of left pericardial agenesis. Given ongoing haemodynamic instability, the patient underwent emergency surgery, which confirmed the complete absence of the left pericardium. The heart was repositioned, and a heterologous pericardial patch was placed. Postoperatively, the patient gradually recovered, requiring short-term renal replacement therapy, and was discharged to rehabilitation.

**Discussion:**

This case highlights a rare but life-threatening presentation of congenital pericardial agenesis. Early recognition and urgent surgical intervention were key to restoring haemodynamic stability. Clinicians should consider this diagnosis when encountering unexplained cardiac displacement or shock, as timely surgical repair can be lifesaving.

Learning points

- Congenital absence of the pericardium, although often asymptomatic, can present as a surgical emergency with cardiogenic shock; early recognition is critical.
- Cardiac levorotation on computed tomography imaging should raise suspicion for pericardial agenesis, especially in unstable patients with unexplained haemodynamic collapse.

## Introduction

Congenital absence of the pericardium is a rare cardiac malformation, occurring in less than 1 in 10 000 people.^1^ It results from a failure in the fusion of the pleuropericardial membranes during embryonic development, most often affecting one side and, more rarely, both sides of the pericardium.^2^ First described in 1559, the condition has typically been discovered incidentally during autopsies or surgeries in asymptomatic patients.^3^ The most serious complication—although extremely rare—is sudden death caused by cardiac herniation or torsion, which occurs mainly in partial pericardial defects, where the remaining pericardial rim may compress or strangulate cardiac structures.^4,5^ Diagnosis is challenging, and many surgeons have never encountered a case. While most patients are asymptomatic, some may present with dyspnoea, chest pain, or associated congenital abnormalities.^1,6,7^

In this case, we present an unusual situation involving an elderly patient in cardiogenic shock, in whom congenital absence of the left pericardium was suspected after a cardiac computed tomography (CT) scan showed cardiac displacement—commonly referred to as heart levorotation. The aim is to highlight the importance of early diagnosis without delaying urgent surgical intervention.

## Summary figure

**Figure:**
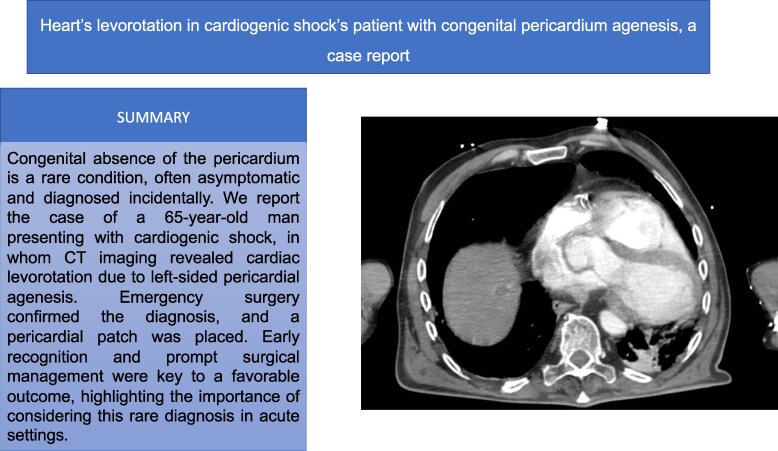


## Case presentation

The patient was a 65-year-old man with several major comorbidities: arterial hypertension, type II diabetes, and left-sided hemiplegia following a stroke many years earlier. A detailed timeline of the patient’s clinical course is provided in *Table 1*. He presented to the emergency department with several days of worsening abdominal pain, general deterioration, and a recent fall at home. On admission, he was conscious, with no new neurological deficits compared to his baseline. On cardiovascular examination, heart sounds were regular with no audible murmurs, no pericardial rub, and no signs of peripheral congestion. His electrocardiogram (ECG) showed diffuse ST-segment elevation. His condition quickly worsened over the following hours, with a progressive decline in consciousness [Glasgow score (GCS) of 5] and haemodynamic instability, requiring norepinephrine up to 2 mg/h. Given the presence of diffuse ST-segment elevation, acute coronary syndrome (ACS) was initially considered. However, the global rather than territorial pattern, together with the absence of chest pain and the severity of metabolic acidosis, made ACS less likely. Because of the patient’s rapidly deteriorating haemodynamic status, no immediate coronary angiography could be performed before surgery. Blood gas analysis revealed severe metabolic lactic acidosis [pH 6.84, lactate 17.5 mmol/L (normal <2 mmol/L)], hyperglycaemia with ketosis, and dark-coloured urine. Hyperkalaemia [7 mmol/L (normal 3.5–5.0 mmol/L)] was treated with insulin, glucose infusion, and bicarbonate.

**Table 1:** Timeline of clinical course

Time	Event
Several days before admission	Progressive abdominal pain, general deterioration
Day 0—Admission	Presentation to the emergency department after a fall; conscious, baseline neurological status
Hours after admission	Rapid clinical deterioration with decreased consciousness (GCS 5) and haemodynamic instability
Day 0	ECG: diffuse ST-segment elevation
Day 0	Blood tests: severe metabolic acidosis, hyperlactatemia, hyperkalaemia
Day 0	Emergency CT scan: cardiac levorotation, suspicion of pericardial defect
Day 0	Transfer to cardiac surgery
Day 0	Emergency surgery: confirmation of complete left pericardial absence; cardiac repositioning and pericardial patch
Postoperative days 1–6	ICU stay: persistent hemodynamic instability, cardiogenic shock, acute kidney injury requiring norepinephrine and haemodialysis
Day 5	Extubation
Day 6	Discontinuation of dialysis
Later course	Transfer to rehabilitation facility
Follow-up	Stable condition, no recurrence of symptoms

**Figure 1:**
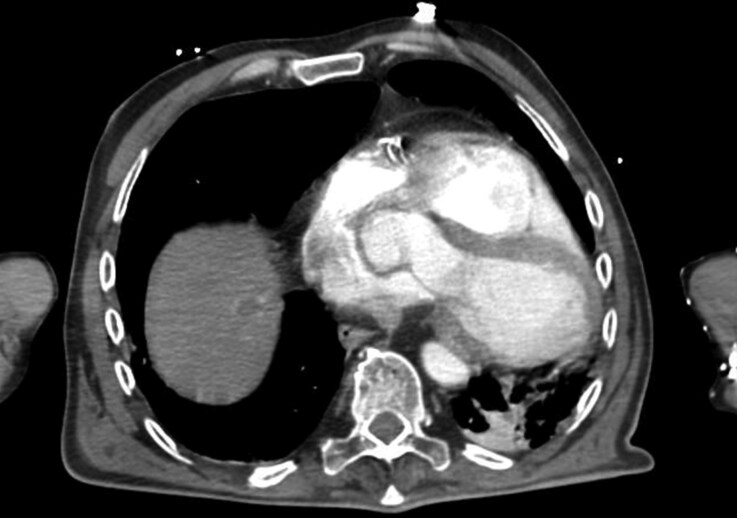
Cardiac CT scan of heart levorotation.

An emergency contrast-enhanced CT scan was performed to rule out aortic dissection, which instead revealed a previously unrecognized displacement of the heart (*Figure 1*). Before surgery, the main working diagnoses included aortic dissection, acute pulmonary embolism, and acute coronary syndrome. Following the CT findings of marked cardiac displacement without vascular injury, differential diagnoses also included diaphragmatic hernia, mediastinal masses, and pericardial rupture or congenital defect. The patient was urgently transferred to the cardiac surgery unit. Arterial and central venous lines were placed, and the patient was intubated. Due to poor haemodynamic tolerance, a median sternotomy was performed, and extracorporeal circulation was initiated via a femoral approach, later switched to central cannulation for improved flow.

**Figure 2:**
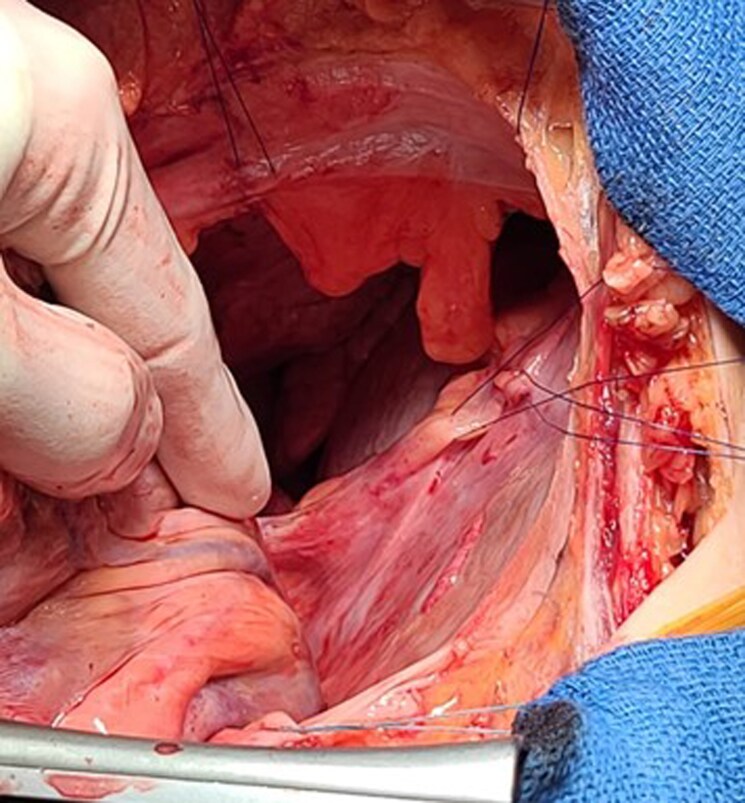
Agenesis of pericardium, presurgical picture.

Surgical exploration confirmed the absence of the left pericardium (*Figure 2*). The heart was displaced into the left hemithorax with counter-clockwise rotation of the right ventricle, and mild deformation of the left pulmonary artery, without clear evidence of vascular obstruction. The surgical team carefully repositioned the heart and anchored it using several sutures. A heterologous pericardial patch was sutured with 3–0 Prolene to reconstruct the left pericardium. The position was confirmed with intraoperative transoesophageal echocardiography.

The patient was transferred to the intensive care unit (ICU) with persistent haemodynamic instability. He developed cardiogenic shock with acute kidney injury, treated with norepinephrine infusion up to 2 mg/h for 5 days and haemodialysis for 6 days. He was extubated after 5 days, and the chest drain was removed on day 4. The outcome was favourable, and he was later transferred to a rehabilitation facility. At follow-up, the patient was fully asymptomatic and had returned to his baseline level of autonomy, living independently with no physical limitations related to the cardiac event.

## Discussion

Congenital absence of the pericardium remains a rare and often under-recognized condition. Left-sided defects account for about 70% of cases, followed by right-sided (17%) and bilateral (9%) forms.^8^ Most patients are asymptomatic, but as this case illustrates, atypical and severe presentations do occur. Early detection and intervention can significantly improve outcomes. Although the patient had a remote history of stroke, no detailed aetiological workup was available in the records. A theoretical link between long-standing cardiac displacement and embolic risk cannot be completely excluded, but such an association remains speculative.

Some authors have described classic chest X-ray findings such as the ‘Snoopy sign,’ characterized by a leftward shift and straightening of the cardiac silhouette.^9^ Echocardiography may not visualize the pericardium directly but can suggest abnormalities, such as unusual septal motion or a swinging heart. CT and cardiac magnetic resonance imaging are more reliable, although even these may not always clearly show the defect.^10^ Therefore, indirect signs such as heart levorotation should prompt further investigation, especially in symptomatic patients, due to the risk of cardiac herniation and sudden death.^3,11^

There is currently no consensus on management, which is primarily based on case reports. Asymptomatic patients usually do not require surgery but should be closely monitored, particularly in cases of partial defects, which may predispose to life-threatening complications such as cardiac herniation or strangulation, especially when combined with other comorbidities (e.g. pulmonary hypertension or congenital cardiac anomalies).^2,12,13^ However, surgery is recommended for symptomatic patients, with good reported outcomes and the potential to prevent fatal complications such as cardiac strangulation.^14,15^ In acute, life-threatening situations like this case, emergency surgical repair is clearly indicated.

This case adds to the existing literature by illustrating an uncommon and dramatic presentation—cardiogenic shock caused by cardiac levorotation due to complete left pericardial agenesis—which required emergency surgery and led to a favourable outcome. The sudden cardiac levorotation may have been triggered by abrupt changes in intrathoracic dynamics—possibly after the fall or abdominal strain—allowing the heart to rotate freely in the absence of pericardial constraints. While multifactorial, the temporal relationship between heart repositioning and haemodynamic improvement supports pericardial agenesis and resultant cardiac rotation as the principal mechanism for shock in this patient. Despite successful repositioning of the heart, the patient remained haemodynamically unstable postoperatively. This deterioration was likely multifactorial, including prolonged preoperative circulatory compromise, severe metabolic acidosis, and possible myocardial stunning following cardiopulmonary bypass. As no single definitive mechanism could be identified, this remains a limitation of the present case.

## Conclusion

Although congenital absence of the pericardium is rare, early recognition is essential, particularly in symptomatic patients. Prompt surgical intervention to reposition the heart and reconstruct the pericardium using a patch can be lifesaving, as demonstrated in this case.

## Data Availability

All data generated or analyzed during this study are included in this study. Further inquiries can be directed to the corresponding author.

## Lead author biography

I am a cardiac surgery resident currently training in Nancy, France. I have a strong interest in rare and challenging clinical cases, which I believe offer valuable learning opportunities and can advance medical knowledge. My passion for cardiac surgery is closely tied to my enthusiasm for clinical research and academic writing. Through the study and publication of unique case reports, I aim to contribute to the continuous improvement of patient care and surgical practice.

## Notes

**Consent:** Written informed consent was obtained from the patient for the publication of this case report and any accompanying images.
